# Cocaine-Induced Time-Dependent Alterations in Cytochrome P450 and Liver Function

**DOI:** 10.3390/ijms24021632

**Published:** 2023-01-13

**Authors:** Joanna Jastrzębska, Władysława Anna Daniel

**Affiliations:** Department of Pharmacokinetics and Drug Metabolism, Maj Institute of Pharmacology, Polish Academy of Sciences, Smętna 12, 31-343 Kraków, Poland

**Keywords:** cocaine, dopaminergic system, addiction, liver, cytochrome P450, hepatotoxicity

## Abstract

Cytochrome P450 is responsible for the metabolism of endogenous substrates, drugs and substances of abuse. The brain and nervous system regulate liver cytochrome P450 via neuroendocrine mechanisms, as shown in rodents. Cocaine exerts its addictive effects through the dopaminergic system, the functioning of which undergoes changes during its continuous use. Therefore, it can be hypothesized that the regulation of cytochrome P450 by cocaine may also alter during the addiction process, cessation and relapse. We analyzed preclinical studies on the mechanisms of the pharmacological action of cocaine, the role of the brain’s dopaminergic system in the neuroendocrine regulation of cytochrome P450 and the in vitro and in vivo effects of cocaine on the cytochrome P450 expression/activity and hepatotoxicity. The results of passive cocaine administration indicate that cocaine affects liver cytochrome P450 enzymes (including those engaged in its own metabolism) via different mechanisms involving the expression of genes encoding cytochrome P450 enzymes and interaction with enzyme proteins. Thus, it may affect its own oxidative metabolism and the metabolism of endogenous substrates and other co-administered drugs and may lead to hepatotoxicity. Its effect depends on the specific cytochrome P450 enzyme affected, cocaine dosage, treatment duration and animal species. However, further complementary studies are needed to find out whether cocaine affects cytochrome P450 via the brain’s dopaminergic system. The knowledge of cocaine’s effect on cytochrome P450 function during the entire addiction process is still incomplete. There is a lack of information on the enzyme expression/activity in animals self-administering cocaine (addicted), in those withdrawn after cocaine self-administration, and during relapse in animals previously addicted; furthermore, there is no such information concerning humans. The subject of cytochrome P450 regulation by cocaine during the addiction process is an open issue, and addressing this topic may help in the treatment of drug abuse patients.

## 1. Introduction

Cocaine is a tropane alkaloid, which has a high potential for addiction. After a short period of drug use, there is a serious risk of dependence development [1]. Cocaine is the second-most widely abused illicit substance worldwide, after cannabis [2]. Globally, there are currently 18 million people who are cocaine addicts [3].

Cocaine addiction is a chronic neurological disorder mainly manifested by compulsive drug-seeking behaviors and high rates of relapse during periods of abstinence [4,5]. Despite many years of research, there are currently no effective medications for cocaine addiction [6]. Cocaine is one of the psychostimulants acting on the central nervous system that increases alertness, heightens arousal and causes behavioral excitement. Cocaine exerts its addictive effects through the dopaminergic system, the functioning of which undergoes changes during continuous cocaine use and after its cessation.

This article aims to assess the potential of cocaine to affect cytochrome P450 during the addiction process. We analyzed the mechanism of pharmacological action of cocaine, the role of the brain’s dopaminergic system in the neuroendocrine regulation of cytochrome P450, and the results of in vivo and in vitro experiments measuring the cytochrome P450 expression/activity and hepatotoxicity after different dosage regiments and times of cocaine administration.

### 1.1. Rewarding System

The action of addictive substances (e.g., cocaine) depends mainly on the reward system of the brain. The reward system is responsible for basic physiological activities, such as food and water intake, sexual behavior, aggression, and motivation [7]. The reward system consists of a number of brain structures with dopaminergic neurotransmission, and the mesolimbic dopaminergic pathway plays an essential role in addiction. The mesolimbic pathway is formed by dopaminergic neurons sending projections from the ventral tegmental area to the nucleus accumbens. The mesolimbic pathway projecting from the ventral tegmental area to the nucleus accumbens plays a major role in the rewarding effects of addictive substances [8], while the mesocortical pathway extending from the ventral tegmental area to the prefrontal cortex is activated during relapses [9].

The most important structure of the reward system, namely the nucleus accumbens—called the ventral striatum—is structurally heterogeneous. In the nucleus accumbens, the shell and core are distinguished in neuroanatomical and functional terms. The nucleus accumbens shell is located medioventrally, and its neurons send projections to the ventral tegmental area and to the basal ganglia. The shell is involved in controlling motivational processes, and emotional, cognitive and motor habits formed during addiction [9]. The nucleus accumbens core is located dorsolaterally, and its neurons send projections mainly to the substantia nigra pars compacta [10]. Activation of the core is associated with the regulation of locomotor activity, food behavior, and autonomous processes, such as blood pressure or heart rate. Additionally, the prefrontal cortex is an important brain structure which is activated by rewarding stimuli. Dopaminergic transmission in the prefrontal cortex is directly related to cognitive processes [11], regulation of emotions [12], working memory [13] and executive functions (planning, moving, attention) [14,15]. This brain structure is also diverse morphologically and functionally. Rewarding stimuli increase the activity of the ventral medial prefrontal cortex [16]. Areas of the brain which are not parts of the reward system but play an important regulatory role in reward processes include the amygdala, hippocampus and lateral habenular nucleus.

Positive reinforcement (a feeling of pleasure) occurs when the extracellular dopamine levels increase at the dopaminergic neuron endings, primarily in the nucleus accumbens [17,18]. Extracellular dopamine level is dependent on other neurotransmitter systems that change the activity of dopamine cells in the ventral tegmental area or at the dopaminergic neuron endings; such dopaminergic neurotransmission modulators include glutamate [19], acetylcholine, ɣ-aminobutyric acid (GABA) [20] and serotonin (5-HT) [21].

The reward system is stimulated by various chemicals that cause a feeling of pleasure and euphoria. One such psychoactive substance responsible for activating the reward system is cocaine.

### 1.2. The Mechanism of Action of Cocaine

Different neurochemical systems are involved in the central and peripheral activity of cocaine. The action on the dopaminergic system is one of the most important effects of this psychostimulant. Cocaine is a dopamine reuptake inhibitor. It binds to the dopamine transporter and blocks the re-uptake of dopamine by the presynaptic neurons. It results in a significant increase in extracellular dopamine levels of up to several orders of magnitude compared to baseline levels [22,23]. Generally, extracellular dopamine levels in the rat brain (the striatum and the nucleus accumbens) return to the baseline levels within 3.5 h after the last (15 mg/kg ip.) cocaine administration [24]. Interestingly, cocaine does not influence the release of dopamine from presynaptic terminals, as is the case with amphetamine. Apart from dopamine, cocaine also inhibits the reuptake of other monoamine neurotransmitters, such as noradrenaline and serotonin. Potency to inhibit neurotransmitter reuptake by cocaine is the highest for serotonin (Ki = 0.14 µM), lower for dopamine (Ki = 0.61 µM) and the lowest for noradrenaline (Ki = 1.60 µM) [20]. However, it is primarily the increase in extracellular dopamine, rather than in serotonin or noradrenaline level, that is directly related to the reinforcing action of cocaine. It is also known from experience with the cocaine self-administration model that the addiction process is mainly dependent on the dopaminergic mesolimbic pathway. Preclinical studies have demonstrated that cocaine analogues of dopamine transporter inhibition (and not of noradrenaline or serotonin transporter inhibition) support the important role of this mechanism (i.e., dopamine transporter inhibition) in the self-administration of cocaine [25].

#### 1.2.1. Chronic Cocaine Administration, Dysfunction of the Dopaminergic System

Chronic administration of cocaine intensifies motor stimulant and stereotypical behavior in rats [26,27]. These behavioral changes are induced by repeated, intermittent exposure to cocaine (sensitization) and are dependent on the dose, dosing schedule, and environmental context. Microdialysis studies have revealed that acute cocaine administration caused tolerance to the increases in the extracellular concentration of dopamine in the nucleus accumbens and dorsolateral striatum (caudate putamen) [28]. Chronic cocaine administration extinguished the acute tolerance observed during early cocaine administration [24]. In addition, chronic cocaine administration resulted in alterations in dopamine receptor densities. In vitro receptor autoradiography technique demonstrated that repeated administration of cocaine (15 mg/kg ip. × 3 per day, for 7 days) caused a transient increase in the D_2_ receptor density in the rostral areas of the nucleus accumbens and caudate putamen of rats [29]. After another 7 days of cocaine administration (14 days in total), the number of D_2_ receptors returned to the basal level. In the case of D_1_ receptor, after 14 days of cocaine administration its quantity increased in the nucleus accumbens without changes in the caudate putamen [30]. Maisonneuve et al. [28] also indicated that the basal levels of extracellular dopamine in the ventromedial and dorsolateral striatum were lower after 14 days of cocaine administration compared to rats that received saline and showed long-term persistence during cocaine withdrawal.

#### 1.2.2. Consequences of Abstinence from Cocaine

During abstinence from cocaine, the mesolimbic system appears to be restored to its normal function but remains in a reactive “incubation” state. Typical abnormalities of the dopaminergic system characteristic of drug withdrawal arise in the seeking and reinstatement phase during the first few days of cocaine withdrawal. Although abstinence can result in apparent normalization of many markers of dopaminergic system function, the system is able to return to its previous state (cocaine use) after one dose of cocaine [31]. Alterations in dopaminergic receptor density, observed during cocaine withdrawal in the nucleus accumbens, are specific and significant. In rats self-administering cocaine (~60 mg/kg/day, 120 infusions), D_1_ receptor surface expression increased in the nucleus accumbens shell on withdrawal day 1, but was normalized on the 45th day of withdrawal. However, at both these points of time (1st and 45th withdrawal day) intracellular D_2_ receptor levels decreased in the nucleus accumbens core and shell. The surface expression of D_2_ receptor was also decreased in the shell, but in the core the decrease occurred only on the 45th day of withdrawal. In the case of D_3_ receptor, changes induced by cocaine on its surface and total expression in the nucleus accumbens core were strictly dependent on time, both were increased on the 45th, but not on the 1st, day of withdrawal [32]. The molecular mechanism of dopamine transporter action was also disturbed after 3 weeks of withdrawal preceded by cocaine self-administration (0.6 mg/kg/infusion, for 10 days). The uptake of dopamine was significantly higher in the caudate putamen and nucleus accumbens and higher surface expression of dopamine transporter was observed, as well [33]. The basal level of dopamine was reduced in the nucleus accumbens after 7 days of discontinued cocaine self-administration, and lasted for a long period of abstinence [34]. The above-presented data obtained in rats support a significant functional dysregulation of the brain’s dopaminergic system during the early stages of abstinence, which usually continues to persist for a very long time, even up to 6 months after the cessation of self-administration.

### 1.3. The Role of the Brain Dopaminergic System in the Regulation of Liver Cytochrome P450

**Figure 1:**
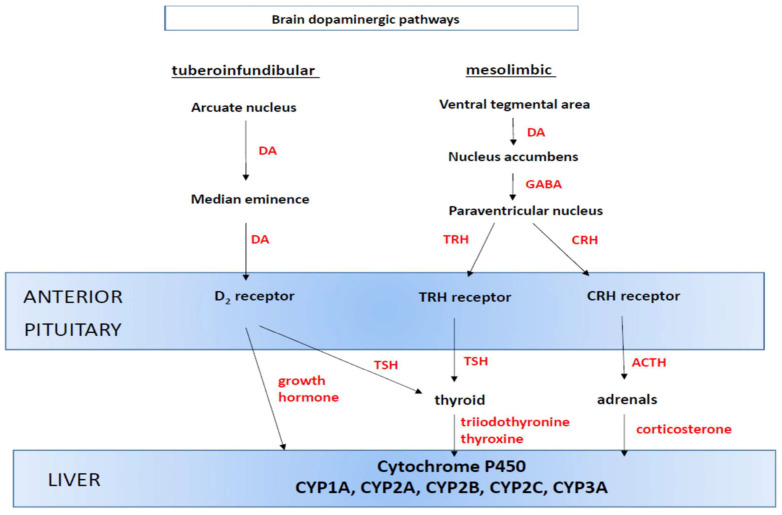
Neuroendocrine regulation of hepatic cytochrome P450 involving the brain dopaminergic system in rats (based on [41]). ACTH, adrenocorticotropic hormone; CRH, corticotropin-releasing hormone; DA, dopamine; GABA, gamma-aminobutyric acid; TRH, thyrotropin-releasing hormone; TSH, thyroid-stimulating hormone. **Mesolimbic pathway:** dopamine released from dopaminergic neurons projecting from the ventral tegmental area to the nucleus accumbens activates D_2_ receptors in the nucleus accumbens, the latter projecting to the hypothalamus containing paraventricular nuclei producing TRH and CRH. TRH and CRH stimulate the release of TSH and ACTH from the anterior pituitary, respectively. TSH stimulates thyroid hormone secretion whereas ACTH activates adrenal corticosterone secretion. **Tuberoinfundibular pathway:** dopaminergic neurons project from the arcuate nuclei of the hypothalamus to the median eminence, from where dopamine is transported via circulation to the anterior pituitary and activates D_2_ receptors present therein, and thus growth hormone and TSH secretion. Growth hormone, thyroxine, triiodothyronine and corticosterone reach the liver via blood circulating and regulate cytochrome expression.

It is feasible that the dopaminergic system can indirectly influence cytochrome P450 expression in the liver by changing the levels of pituitary hormones and/or cytokines [35,36,37]. Microcellular neurons in the arcuate nuclei of the hypothalamus liberate growth hormone-releasing hormone (GHRH), while microcellular neurons in the paraventricular nuclei of the hypothalamus produce corticotropin-releasing hormone (CRH), thyrotropin-releasing hormone (TRH) and growth hormone-inhibiting hormone (somatostatin) [36,38]. Furthermore, a few neuronal projections from the nucleus accumbens septi to the hypothalamus (e.g., to the paraventricular nuclei) may be of functional significance for neuroendocrine regulation [39,40]. This means that changes in functioning of the dopaminergic system (i.e., the tuberoinfundibular and mesolimbic pathway) can be crucial for pituitary hormone secretion and the physiological regulation of cytochrome P450 (Figure 1).

Wójcikowski and Daniel [41,42] showed that the activation of these brain dopaminergic pathways was significant to the regulation of liver cytochrome P450, namely CYP1A, CYP2B, CYP2C11 and CYP3A in rats (Table 1). Activation of the tuberoinfundibular pathway leading to the stimulation of pituitary dopaminergic receptors (by D_1_/D_2_ receptor agonist) caused a significant increase in the activities and protein levels of CYP2B, CYP2C11 and CYP3A, accompanied by a substantial increase in growth hormones and a decrease in T3 concentration in the blood plasma. On the other hand, activation of the mesolimbic pathway by stimulation of dopaminergic receptors in the nucleus accumbens by apomorphine (D_1_/D_2_ receptor agonist), amphetamine (releasing dopamine and inhibiting dopamine re-uptake) or quinpirole (D_2_ receptor agonist) resulted in the increased activity and protein levels of CYP3A, accompanied by a simultaneous large increase in the concentration of corticosterone and a moderate decrease in that of T_3_. Moreover, the activation of the tuberoinfundibular or mesolimbic pathways considerably decreased the activity and protein levels of CYP1A [42]. Damage to these dopaminergic pathways produced opposite effects in rats [43]. Lesion of the tuberoinfundibular pathway decreased the activity and protein levels of CYP2B, CYP2C11 and CYP3A. Lesion of the mesolimbic pathway reduced the activity and protein level of CYP3A. In contrast, the CYP1A activity was significantly elevated after lesion of the tuberoinfundibular or the mesolimbic pathway. Damage to those dopaminergic pathways did not affect the activities of CYP2A, CYP2C6 and CYP2D, whose regulation is less susceptible to hormonal changes. However, lesion of the nigrostriatal pathway (not affecting central hormonal system) did not affect any of the studied CYP enzymes [43]. Apart from dopaminergic system, other brain neurotransmitter systems (noradrenergic, serotonergic and glutamatergic) also contribute to the central neuroendocrine regulation of liver cytochrome P450 [44].

Considering the role of the brain’s dopaminergic system in the physiological regulation of liver cytochrome P450 and in cocaine addiction (shown in independent animal studies), the purpose of this review is to analyze the published experimental data in the context of indirect regulation through the dopaminergic system of hepatic cytochrome P450 by cocaine at different stages and conditions of drug administration. Since the functioning of the brain’s dopaminergic system changes during the cocaine addiction process, it may be expected that the regulation of cytochrome P450 (enzyme expression/activity) also undergoes respective modifications.

## 2. Cytochrome P450

Cytochrome P450 (CYP) is a huge family of monooxygenases with hemoprotein structures. CYP enzymes are responsible for the oxidative metabolism of a vast variety of both exogenous and endogenous substances [45,46]. The spectral properties of these enzymes were the reason for introducing the term ‘cytochrome P450′ [47,48]. In the name ‘cytochrome P450’, cytochrome stands for a hemoprotein, P for pigment and 450 reflects the absorption n peak of its reduced complex with carbon monoxide at 450 nm. CYPs have been classified into eighteen families and 57 cytochrome subfamilies. They were distinguished based on the similarity of their amino acid sequences [49]. Each family (designated by an Arabic number) comprises sequences that are 40% or more identical at the amino acid level. Each subfamily includes sequences that are 60% or more identical (designated by a capital letter), and individual enzymes (and their genes) are arbitrarily numbered [50]. The first three families CYP1–CYP3 catalyze steroid metabolism and they are the main enzymes that metabolize drugs and other xenobiotics [51]. Many of the psychopharmacological drugs acting on the central nervous system, such as antidepressants and antipsychotics, are metabolized by the CYP2 and CYP3A families [46,52]; the same applies to drugs of abuse, such as ethanol and cocaine, and some endogenous neurochemicals including steroids, 5-HT and DA [53,54,55]. The higher CYP family numbers are primarily responsible for the metabolism of endogenous compounds. The highest level of CYP proteins is found in the liver, but these are also present in almost all body tissues [56]. Most of the hepatic CYP enzymes are detected in the smooth endoplasmic reticulum, but also in the mitochondrial and nuclear membranes. In the literature, 85 eukaryotic (including vertebrates, invertebrates, fungi, and plants) and 20 prokaryotic CYP genes have been described. Fourteen of seventy-four known gene families occur in mammals [45,57,58]. Around 57 different CYP genes and 58 pseudogenes have been described in humans.

As mentioned above, the enzymes in the families CYP1–CYP3 (CYP1A2, 2C9, 2C19, 2D6, 2E1 and 3A4) are especially active in the hepatic metabolism of xenobiotics, and the remaining families have significant endogenous functions [46,59,60,61].

Addictive substances, such as cocaine, nicotine or amphetamine are substrates or inhibitors of some CYP enzymes, and their metabolism has a critical role in pharmacological and toxicological consequences during exposure to them. Previous research has determined, to a large extent, the role of metabolic enzymes in susceptibility to drug dependence, metabolic tolerance, and variability in the toxic consequences of exposure to those xenobiotics.

## 3. Cocaine Metabolism and Hepatotoxicity

The duration of action of cocaine is relatively short. The peak increase in heart rate occurs about 60 min after the administration and declines thereafter [62]. This is due to the rapid hydrolysis of cocaine into two major deesterified metabolites: (1) ecgonine methyl ester produced in the liver by carboxylesterase type 2 (hCE2) and in plasma through pseudocholinesterase (PchE), more specifically butyrylcholinesterase [63], and (2) benzoylecgonine also formed in the liver by carboxylesterase type 1 (hCE1) (Figure 1). They represent 95% of the metabolic products of cocaine excreted in the urine [64,65,66]. These metabolites, even when administered in high doses, are not active as stimulants [67,68]. Additionally, simultaneous consumption of cocaine and alcohol leads to a different metabolic path via transesterification between cocaine and ethanol to form cocaethylene, and subsequent hydrolysis through hCE1. After intravenous administration, the half-life of cocaine in the rapid disposition phase is about 10 min, while in the elimination phase 50 to 80 min. Therefore, stimulation by cocaine is limited by the speed of its hydrolysis to inactive metabolites [69].

The thermal breakdown of the inhaled form of cocaine (“crack”) leads to ecgonidine methyl ester [70,71]. Next, hCE1 hydrolyses ecgonidine methyl ester into ecgonidine. If the hydrolysis is catalyzed in the presence of alcohol, ecgonidine ethyl ester is produced [72].

**Figure 2:**
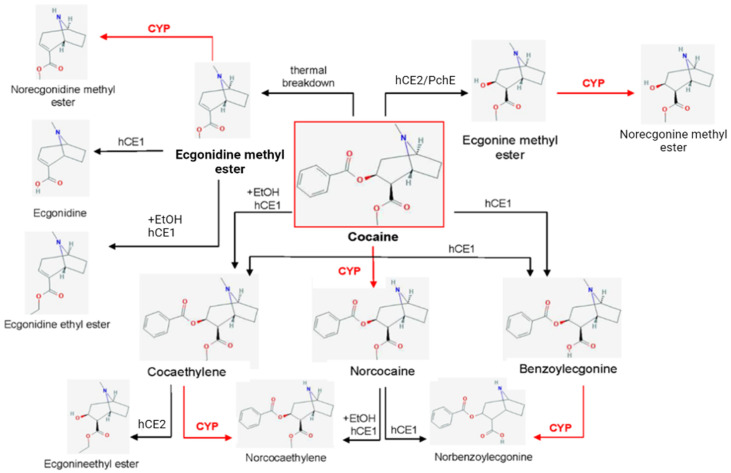
Scheme representing cocaine metabolism pathways with particular reference to the role of cytochrome 450 (CYP) (based on [77]. EtOH—ethanol; hCE1—human carboxylesterase type 1, hCE2—human carboxylesterase type 2, PchE—pseudocholinesterase.

The remaining 5% of cocaine reaching the liver is bioactivated into norcocaine via cytochrome P450 [73,74,75]. Cytochrome P450 is also responsible for the N-demethylation of benzoylecgonine into norbenzoylecgonine, cocaethylene into norcocaethylene, ecgonine methyl ester into norecgonine methyl ester and ecgonidine methyl ester into norecgonidine methyl ester [76] (Figure 2).

The preclinical studies on animals showed that cocaine is N-demethylated by CYP2B enzymes in rats [78] and by CYP3A in mice [79,80]. Using the microsomal fractions from mouse livers, kinetic studies have proven that multiple enzymes catalyze cocaine N-demethylation, with a high Km component sensitive to the specific inhibitors of CYP3A enzymes, such as triacetyl-oleandomycin and gestodene [79]. Rat CYP2B2 and dog CYP2B11 are the main N-demethylases of cocaine in these species, but only in rats does the CYP2B enzyme catalyse further oxidation of norcocaine [81]. Results from the human liver microsomal fraction and lymphoblasts expressing CYP3A4 confirm that human CYP3A4 mediates cocaine N-demethylation, but does not catalyze norcocaine N-hydroxylation [67]. In contrast to rats and dogs, the human CYP2B enzyme (CYP2B6) probably is not involved in cocaine N-demethylation [79]. Immunoinhibition studies of Powers and Shuster [82] showed that CYP2A5 was the major isoform responsible for norcocaine N-hydroxylation in mice.

Cocaine damages the liver and leads to necrosis in animals [83] and humans [84,85,86,87]. Norcocaine and additional secondary oxidative metabolites are mainly involved in these injuries. These metabolites are involved in the exhaustion of antioxidant defenses, disruption of cellular activity and activation of redox cycles, and subsequently generate reactive oxygen species (ROS), leading to oxidative stress. Cocaine, as opposed to other xenobiotics, evokes zone necrosis of the liver, depending on the dose, sex and enzyme induction or inhibition [85]. Liver impairment evoked by cocaine usually goes along with other organ impairments [88].

As mentioned above, there are large species variations in the CYP enzymes involved in cocaine metabolism: CYP2A, CYP2B and CYP3A in mice [79,89,90], CYP2B and CYP3A in rats [78,81,91], and only CYP3A, peculiarly CYP3A4, in humans [75,79,92]. Hence, there is a wide inter-species divergence in the degree of liver damage induced by cocaine. Mice are the most vulnerable species, followed by rats and humans (as the least susceptible species) [93].

The supposed mechanism of hepatotoxicity of cocaine is based on down-regulation of the glutathione peroxidase (GPx) and catalase gene expression. After acute cocaine administration in rats, higher ROS production was observed, which resulted in elevated peroxide levels and manganese superoxide dismutase activity, but catalase and GPx activity were reduced. The decreased catalase and GPx activity contributes to H_2_O_2_ accumulation, HO˙ formation and finally to oxidative stress-related tissue damage [94]. Mitochondria are probably a particular target for the hepatotoxic effects of cocaine. Several studies have shown depressed mitochondrial respiration and increased ROS levels in these organelles after cocaine use [95,96,97,98]. The consequence of mitochondrial respiration impairment is depletion of intracellular adenosine triphosphate and activation of apoptotic pathway, foremost cytochrome c release and caspase-3 induction [78,89,94,96,97,98,99]. Interestingly, the increase in mitochondrial ROS production after cocaine administration in mice was not as significant as in rats [100], which may indicate that cocaine has significant effects also on other cellular organelles, such as the endoplasmic reticulum, where oxidative metabolism of cocaine takes place [95].

It has been shown that administering cocaine in a dose of 30 mg/kg/day ip. for 3 days did not cause liver damage in mice or rats [82], but a dose of 60 mg/kg ip. administered for 3 days induced severe hepatocellular necrosis in the pericentral zone of mouse livers [101]. After 10–14 days of repeated cocaine administration (60 mg/kg ip.) regenerative changes in hepatocytes were observed [101].

Summarizing, 5% of cocaine in the body is bioactivated into norcocaine via cytochrome P450 in the liver. Cocaine is mainly N-demethylated by CYP2B enzymes in rats and by CYP3A in mice. In humans, CYP3A4 mediates cocaine N-demethylation, although it does not catalyze norcocaine N-hydroxylation. Moreover, in humans CYP2B is not involved in cocaine N-demethylation. In addition, susceptibility to cocaine-induced liver damage is highly species-dependent, being the highest in mice.

## 4. The Effects of Cytochrome P450 Inducers on Cocaine Metabolism and Hepatotoxicity

Metabolic events contributing to cocaine-induced hepatotoxicity have been studied with several cytochrome inducers. Mice pretreated for 4 days with dexamethasone (100 mg/kg ip.) before administration of cocaine (60 mg/kg ip.) showed an increased liver CYP3A and CYP2B content and oxidative cocaine metabolism, although they were not more susceptible to cocaine hepatotoxicity. However, mice treated with phenobarbital (the same pattern of administration) exhibited comparable increases in those CYP enzymes but were susceptible to cocaine hepatotoxicity. The authors suggest that the lack of susceptibility of dexamethasone-pretreated mice is probably due to a significant rise in cocaine esterase action. No increase in liver CYP3A activity or the oxidative metabolism of cocaine was observed in animals treated with phencyclidine, but they were quite susceptible to cocaine hepatotoxicity [90].

Pellinen et al. [79] also showed that the administration of phenobarbital (3 × 100 mg/kg ip.) to DBA/2/KUO (D2) male mice increased cocaine N-demethylase activity by elevating the CYP3A and CYP2B activity, as well as induced norcocaine metabolism to a hepatotoxic radical. Surprisingly, they did not observe enhanced toxicity of cocaine after the CYP2B and CYP3A inducer pregnenolone-16-carbonitrile (3 × 50 mg/kg ip.), as has been reported for phenobarbital. They explain this result by an increased cocaine N-demethylase activity, associated with CYP3A induction, which may not increase or even reduce the production of the final toxic metabolite by lowering the content/expression of the currently unidentified enzymes responsible for the final stage of activation [79].

Aoki et al. [89] in their studies also underlined the important correlation between cocaine N-demethylation and CYP3A in ICR mice, although the research was conducted on female mice in which single doses of cocaine (45 mg/kg ip.) did not cause significant hepatotoxicity. However, their results do not confirm that CYP1A, CYP2B and CYP3A are essential for hepatotoxicity in female mice. CYP1A and CYP2B were induced by β-naphthoflavone, but this did not affect cocaine-induced hepatotoxicity and cocaine N-demethylase activity. Contrary to the suggestion of Pellinen et al. [79], hepatotoxicity caused by cocaine was potentiated by β-ionone without CYP3A induction almost to the same degree as by phenobarbital. However, cocaine N-demethylation was inhibited by the CYP2A-specific inhibitor 8-methoxypsoralen and pretreatment with 8-methoxypsoralen lead to a marked inhibition of the cocaine hepatotoxicity in phenobarbital-treated mice. Therefore, it seems possible that cocaine-induced hepatotoxicity in female mice may be partly mediated by CYP2A, the enzyme participating in cocaine N-demethylation [89].

## 5. The Impact of Cocaine on the Liver Cytochrome P450 in Preclinical Research in Animal Models

Numerous animal studies have demonstrated changes in the activity and/or expression of cytochrome P450 in the liver after cocaine administration using different research protocols and in combination with other substances (Table 2).

### 5.1. Influence of Acute Cocaine Administration on Cytochrome P450 Enzymes

Konstandi and Lang [102], similar to Pellinen et al. [80,101], have shown that a single dose of cocaine (60 mg/kg ip.) increases the CYP2A5 activity, but contrary to Pellinen et al. [80] they revealed that this dose of cocaine enhanced the levels of CYP2A5 protein and mRNA in mice. These discrepancies are very difficult to explain, because researchers used the same experimental procedures and mice from the same strain (Male DBA/2/KUO (D2)).

On the other hand, parallel studies carried out on two strains of male mice (DBA/2/KUO (D2) and C57BL/6N/KUO (B6)) that received a single dose of cocaine (60 mg/kg ip.) showed a 4-fold increase in the CYP2A5 activity in DBA/2/KUO (D2), but no change in C57BL/6N/KUO (B6) mice. Similarly, a 2-fold increase in the CYP2A4 activity was observed in the DBA/2/KUO (D2) mice only. Nevertheless, the total CYP levels did not relevantly differ between the two strains. These two strains did not show any significant differences in other CYP-catalyzed reactions, either [103].

Cocaine administered in single doses of 7.5, 15, 30, 60 or 80 mg/kg ip. to DBA/2KUO (D2) male mice increased the CYP2A4/5 activity up to a dose of 60 mg/kg, where maximum induction (3.8-fold) occurred, and a dose-dependent increase of CYP2A4/5 protein levels was also observed. On the other hand, the mRNA level at the initial dose (15 mg/kg, ip.) was lowered and it increased significantly from a dose of 30 to 80 mg/kg. This increase in the mRNA level was clearly greater (10-fold) compared with the enhancement of CYP2A4/5 activity and immunoreactivity of the CYP2A4/5 protein (4-fold). The authors suggest that this is a consequence of ongoing gene transcription or stabilization of the message, but a block at the level of translation or enzyme function is also possible. However, in this experimental scheme, no changes in CYP2B10 activity or in the content of CYP2B10 protein and mRNA were observed [80].

### 5.2. The Effects of Repeated or Chronic Cocaine Administration on Cytochrome P450 Enzymes

Vitcheva and Mitcheva [104] demonstrated that cocaine (15 mg/kg ip.) administered for 5 days (once a day) significantly reduced the total content of cytochrome P450 in rats. The authors also used mixed applications of cocaine with nifedipine (5 mg/kg ip.), the latter being also a substrate for CYP3A. In the combination group, changes in cytochrome P450 quantity showed that these two compounds mutually abolished their effects on the enzyme, i.e., a decrease caused by cocaine and an increase caused by nifedipine (Table 2).

Repeated administration of cocaine (30 mg/kg/day ip. for 3 days) did not induce liver damage in two strains of mice (B6AF1/J male/female and Balb/cBy male) or male Wistar rats, as measured by the leakage of glutamic-pyruvic transaminase into the bloodstream. Likewise, the total CYP content was similar to the control in these animals [82]. The activity and protein levels of CYP3A, CYP2E and CYP2D9 were unaffected by a three-day cocaine administration (30 mg/kg). Detailed studies showed that the CYP2A4/5 protein level and activity increased with each subsequent dose (30 mg/kg ip.) of cocaine (protein: 23%, 40% and 44%; activity: 25%, 40% and 80%) in male and female B6AF1/J mice. Similarly, in male Balb/cBy mice, the authors observed even higher activity and protein levels of CYP2A4/5, but they were not statistically significant when compared to the strain B6AF1/J. In addition, daily administration of cocaine (30 mg/kg ip.) increased the CYP2B10 protein level by 5% above the control after the first dose, by 31% after the second dose, and 57% after the third dose in male B6AF1/J mice (Table 3). Liver microsomes from female B6AF1/J mice had a significantly higher protein levels of this enzyme than microsomes from males. Accordingly, the CYP2B10 activity in B6AF1/J mice increased to 142% of the control after the third dose of cocaine (30 mg/kg ip.) [82]. Repeated cocaine administration (30 mg/kg/day ip. For 3 days) increased the CYP2A5-catalyzed N-hydroxylation of norcocaine, which indicates that chronic cocaine can induce its own metabolism [82].

In another time-response experiment, a single cocaine dose (60 mg/kg ip.) was given at intervals of 4 h, 12 h, 24 h, 72 h (for 3 days at 24 h intervals) and 120 h (for 5 days at 24 h intervals) to DBA/2/KUO (D2) male mice, and the enzymatic studies were carried out [80]. Four hours after administering the cocaine, the CYP2A4/5 activity and protein and mRNA contents were slightly decreased. After 12 h, the mRNA and protein levels of these enzymes were markedly lowered, but after 24 h there was a maximum induction followed by a rapid and consistent decrease in the CYP2A4/5 activity, protein and mRNA. Importantly, five days after cocaine administration, the activity of CYP2B10 was 5.8-fold higher and protein levels increased by approximately 4-fold. This change of CYP2B10 may indicate that cocaine has the ability to enhance its own metabolism [80]. In contrast, the content of CYP2El and CYP1A1/2 protein and their mRNAs were decreased at each time point in relation to the respective starting values.

However, during chronic cocaine administration (60 mg/kg, ip. for 14 days) to DBA/2/KUO (D2) male mice, a maximum 14-fold increase in the CYP2B10 protein level was observed on the 7th day of the experiment, while the enzyme activity increased 2-fold on day 5 and gradually increased until day 14 [101]. Concerning CYP2A4/5, both protein content and activity significantly increased on day 1 and considerably decreased on day 3, 5 and 7. However, on day 10 and 14 of the experiment, both protein levels and the activity of CYP2A4/5 escalated. Likewise, a slight increase in the CYP2C protein level on day 1 was followed by a decrease from days 3 to 7 and a further increase (2-fold) from days 10 to 14. The CYP2C activity (measured as the rate of benzphetamine N-demethylation) decreased in the first three days but increased significantly from the 4th day onwards with each subsequent dose of cocaine. Changes in the CYP1A protein level were the same as in the case of CYP2C. In contrast, the CYP1A activity was reduced by 50% on day 3, 5 and 7, but returned to control levels on day 10 and 14. The study also showed that from the seventh daily dose of cocaine, the CYP3A protein level rose 2- to 6-fold by the end of the trial. The CYP3A activity temporarily decreased after three doses of cocaine, and then increased from about 2-fold after 7 days to 5-fold after 14 daily cocaine injections (Table 4).

The authors stress that repeated cocaine injections are associated with multiple liver foci of necrosis, followed by regeneration, resulting in the appearance of young hepatocytes with high enzymatic activity, and hence, in an enhanced expression of the mentioned CYP enzymes in rodents [101]. The above results suggest that cocaine accelerates its own CYP-dependent metabolism via CYP3A-catalyzed production of norcocaine, whereas CYP2B10 (induced by cocaine) has a minor role in cocaine hepatotoxicity.

Summing up, cocaine affects cytochrome P450 expression and activity in the liver, mainly CYP2A and CYP2B, but also other CYP enzymes (CYP1A/2C/2E/3A) via different mechanisms involving the regulation of transcription and interaction with enzyme proteins. Its effect depends on the particular CYP enzyme, cocaine dosage, treatment duration and animal species. It is also interesting to note that changes in the expression/activity of CYP enzymes, observed in rodents after repeated/chronic treatment with cocaine (i.e., a decrease in CYP1A and an increase in CYP2B, CYP2C and CYP3A), are consistent with those found in rats after the activation of the brain’s dopaminergic system, i.e., the mesolimbic and tuberoinfundibular pathways, by selective dopaminergic agonists (Table 1 and Table 4). It may suggest that, in rodents, cocaine affects liver cytochrome P450 via its central dopaminergic mechanism and neuroendocrine pathway (Figure 1), though other mechanisms operating in the liver may also be involved. However, these are two independent observations (the effects of cocaine and of dopaminergic agonists on cytochrome P450), so the involvement of brain’s dopaminergic system in the effect of cocaine on liver cytochrome P450 in rodents is hypothetical at this stage of studies. Moreover, the effects of cocaine on liver cytochrome P450 may be different after its longer administration (simulating addiction state) or after drug withdrawal (resembling abstinence state), i.e., when the functioning of the brain’s dopaminergic system and, in turn, regulation of physiological processes change. It is also important to note that the presented results are preclinical and obtained in animals, which cannot be easily translated on humans.

## 6. Conclusions

Cocaine, one of the most frequently abused psychostimulants, exerts its action both in the central nervous system (mainly via dopaminergic system) and in peripheral organs, such as the liver (affecting cytochrome P450 functioning), as shown in the studies carried out in rodents. It has been demonstrated in rats that disorders in the functioning of the tuberoinfundibular and mesolimbic pathways of the brain’s dopaminergic system have an impact on the liver cytochrome P450 expression (CYP1A, CYP2B, CYP2C and CYP3A). Based on the available preclinical data, it is hypothesized that cocaine may affect hepatic cytochrome P450 expression and liver function (including hepatotoxicity) by acting locally or indirectly through the central nervous system, mainly the dopaminergic system. Cocaine-mediated regulation of liver cytochrome P450 may involve the activation of D_2_ receptors in the nucleus accumbens and the pituitary gland. However, the basic mechanisms of cocaine action on liver cytochrome P450 do not reflect all possible changes in enzyme functioning during the entire duration of the addiction process, since the response of the dopaminergic system and hepatotoxic processes vary depending on the stage of treatment or its cessation. In the available literature, only the effects of passive acute and chronic cocaine administration on cytochrome P450 have been studied in rodents, and investigations mainly focused on the direct cocaine action in hepatocytes. Thus, the picture of cocaine’s effects through the entire addiction process in the context of CYP function is incomplete. There is a lack of information on the expression and activity of CYP enzymes in animals self-administering cocaine (addicted) and in those withdrawn after cocaine self-administration. It would also be interesting to observe the results in animals previously addicted during relapse to cocaine self-administration, because disorders in the density of D_1_ and D_2_ receptors (especially in the rewarding system), changes in extracellular dopamine level, and the number of dopamine transporters are irregular in the long-term and can persist for months of abstinence. The cocaine-induced modifications in the dopaminergic system remain dormant during abstinence, but the system easily returns to the state of addiction. It is possible that alterations in cytochrome P450 enzymes are also long-lasting, as suggested by our preliminary results [105], and sensitive to cocaine re-administration. Moreover, there is a lack of information on possible changes in particular brain CYP enzymes involved in the metabolism of endogenous neuroactive substrates (cholesterol, neurosteroids, neurotransmitters) during cocaine treatment and the whole addiction process.

Thus, further complementary studies are necessary to determine whether cocaine can affect cytochrome P450 via the brain’s dopaminergic system in the studied animal species, and whether similar alterations in the cytochrome P450 function occur in humans during cocaine addiction. The results of such experiments would have a great pharmacological significance in relation to the treatment of cocaine-dependent patients. If cocaine alters liver cytochrome P450 expression/activity in humans, it might affect its own oxidative metabolism and the metabolism of endogenous substrates and other co-administered drugs depending on the stage of addiction process. The subject of regulation of cytochrome P450 by cocaine in the liver and brain is an open issue and addressing this topic may pave new ways to the treatment of drug abuse patients.

## Figures and Tables

**Table 1:** Impact of the activation of brain dopaminergic pathways by different ligands of dopaminergic receptors on the activity and protein levels of cytochrome P450 (CYP) isoforms in rat liver microsomes.

CYP	Tuberoinfundibular Pathway		Mesolimbic Pathway					
	Dopamine (D_1_/D_2_ Receptor)		Apomorphine (D_1_/D_2_ Receptor)		Amphetamine (D_1_/D_2_ Receptor)		Quinpirole (D_2_ Receptor)	
	Activity	Protein Level	Activity	Protein Level	Activity	Protein Level	Activity	Protein Level
CYP1A	** 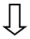 **	** 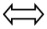 **	** 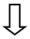 **	** 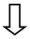 **	** 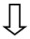 **	** 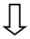 **	** 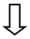 **	** 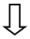 **
CYP2A	** 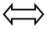 **	**NT**	** 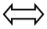 **	**NT**	** 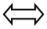 **	**NT**	** 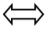 **	**NT**
CYP2B	** 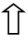 **	** 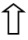 **	** 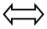 **	**NT**	** 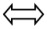 **	**NT**	** 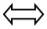 **	**NT**
CYP2C11	** 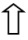 **	** 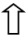 **	** 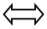 **	**NT**	** 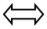 **	**NT**	** 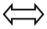 **	**NT**
CYP3A	** 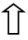 **	** 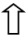 **	** 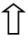 **	** 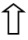 **	** 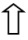 **	** 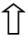 **	** 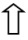 **	** 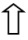 **

Based on [42]. 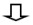 decrease, 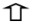 increase, 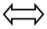 unchanged, NT not tested.

**Table 2:** Effects of cocaine or combinations of cocaine and CYP inducers on the CYP expression and activity in selected animal species.

Species	Injection/Duration of Treatment	Dose (mg/kg)	Time of Last Injection	Effect	Publication
DBA/2/KUO (D2) male	ip. a single dose	60	24 h	CYP2A4/5—increase in activity, protein level and mRNA	[102]
DBA/2/KUO (D2) male	ip. a single dose	60	24 h	CYP2A4/5—increase in activity and protein level	[103]
C57BL/6N/KUO (B6) male	ip. a single dose	60	24 h	CYP2A4/5—no change in activity and protein level	[103]
DBA/2/KUO (D2) male	ip. a single dose	7.5,15, 30, 60, 80	24 h	*CYP2A4/5*—mRNA level lowered at dose 7.5 and 15 mg/kg, but increased significantly from dose 30 to 80 mg/kg CYP2A4/5—maximum induction at dose 60 mg/kg CYP2B10—protein and mRNA unchanged	[80]
Wistar rat male	ip. a single dose for 5 days	15	24 h	quantitative decrease in total CYP (spectrophotometry)	[104]
B6AF1/J male	ip. a single dose for 3 days	30	18 h	no liver damage CYP2A4/5—maximum activity and protein level after 3 days CYP3A, 2E, 2D9—no change in activity and protein level CYP2B10—no change in activity and maximum protein level after 3 days	[82]
B6AF1/J female	ip. a single dose for 3 days	30	18 h	no liver damage CYP2A4/5—maximum activity and protein level after 3 days CYP3A, 2E, 2D9—no change in activity and protein level CYP2B10—maximum activity and protein level after 3 days	[82]
Balb/cBy male	ip. a single dose for 3 days	30	18 h	no liver damage CYP2A4/5—maximum activity and protein level after 3 days CYP3A, 2E, 2D9—no change in activity and protein level CYP2B10—activity, protein level and mRNA unchanged	[82]
Wistar rats male	ip. a single dose for 3 days	30	18 h	no liver damage CYP3A, 2E, 2D9—no change in activity and protein level CYP2A4/5—protein level and mRNA unchanged CYP2B10—protein level and mRNA unchanged	[82]
DBA/2/KUO (D2) male	ip, 4, 12, 24, 72 and 120 h exposure	60	24 h	*CYP2A4/5* maximum induction after 24 h CYP2B10 maximum activity after 5 days CYP2E1, CYP1A1/2 protein and mRNA level decrease in subsequent points of time	[80]
DBA/2/KUO (D2) male	ip. a single dose for 14 day	60	24 h	CYP2A4/5 protein level and activity increased on day 1, decreased on days 3, 5 and 7, increased again on days 10 and 14 CYP2B10 maximum increase in protein level on 7 and activity on day 5 CYP2C protein level increased on day 1, decreased on days 3, 5 and 7, increased again on days 10 to 14 CYP2C activity decreased in the first 3 days, increase from 4 to 14 days CYP1A protein level increased on day 1, decreased on days 3, 5 and 7, increased again on days 10 to 14 CYP1A activity decreased on days 3, 5 and 7, returned to control value on days 10–14 CYP3A protein level rose from day 7 to 14, activity decreased on days 3–7, again increased on days 7–14	[101]
Male CF-1 mice	pretreatment with P450 inducers for 4 days, cocaine ip. a single dose	60	20 h	Increased CYP3A and CYP2B activity by phenobarbital and enhanced susceptibility to cocaine hepatotoxicity Increased CYP3A and CYP2B activity by dexamethasone, but no change in cocaine hepatotoxicity	[90]
Male DBA/2/KUO (D2)	pretreatment with P450 inducers for 3 days, cocaine ip. a single dose	25 or 60	16 h	Increased CYP3A and CYP2B activity by phenobarbital and enhanced cocaine hepatotoxicity Increased CYP3A and CYP2B activity by pregnenolone-16-carbonitrile, but no change in cocaine hepatotoxicity	[80]
Female ICR mice	pretreatment with P450 inducers or inhibitors for 3 days, cocaine ip. a single dose	35–45	24 or 48 h	Increased CYP1A and CYP2B activity by β-naphthoflavone, without enhancing cocaine hepatotoxicity No change in CYP3A activity by β-ionone, but potentiation of cocaine hepatotoxicity CYP2A inhibition by 8-methoxypsoralen reduced cocaine hepatotoxicity in phenobarbital-treated mice	[89]

**Table 3:** The effect of repeated administrations of cocaine (30 mg/kg ip. for 3 days) on protein levels and activity of selected cytochrome P450 (CYP) enzymes in animal livers.

Species	Cytochrome P450	Cocaine Day 1	Cocaine Day 2	Cocaine Day 3
	CYP2A			
B6AF1/J male		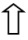	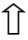	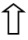
B6AF1/J female		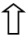	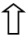	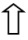
Balb/cBy male		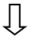	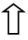	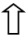
Wistar rats male		N.D.	N.D.	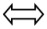
	CYP2B			
B6AF1/J male		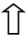	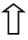	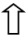
B6AF1/J female		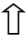	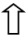	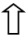
Balb/cBy male		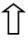	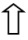	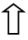
Wistar rats male		N.D.	N.D.	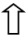

Based on [82]. Cocaine HCl (30 mg/kg ip.) was injected intraperitoneally for 1, 2 or 3 days. The drug was dissolved in 0.9% NaCl (saline) and injected in a volume of 0.1 mL/10 g body weight. Control animals were injected with equal volumes of saline. At 16–18 h after the final drug administration animals were killed. The effects of cocaine on the selected CYP protein levels and activities were compared with those of control (saline) group. 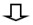 decrease, 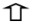 increase, 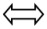 unchanged, N.D. not detected.

**Table 4:** The effect of chronic administrations of cocaine (60 mg/kg ip. for 14 days) on total cytochrome P450 (CYP) content and activity of selected CYP enzymes in DBA/2/KUO (D2) male mice.

Days of Treatment	CYP (Total)	CYP3A	CYP2B	CYP2A	CYP2C	CYP1A
1	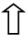	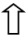	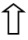	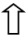	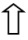	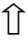
3	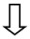	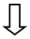	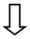	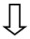	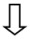	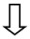
5	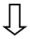	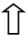	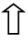	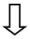	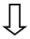	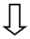
7	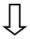	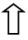	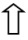	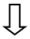	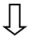	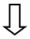
10	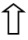	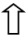	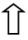	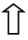	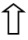	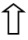
14	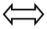	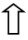	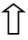	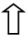	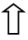	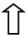

Based on [101]. Cocaine HCl (60 mg/kg) was injected intraperitoneally for 1 to 14 days, once a day at 9 am. The drug was dissolved in 0.9% NaCl (saline) and injected in a volume of 0.1 mL/20 g body weight. Control animals were injected with equal volumes of saline. At 24 h after the final drug administration animals were killed. 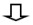 decrease, 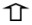 increase, 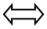 unchanged.

## Data Availability

Not applicable.
